# Day-by-day symptom relief after corticosteroid injection for trigger digit: a randomized controlled study of two techniques

**DOI:** 10.1177/17531934231177422

**Published:** 2023-05-22

**Authors:** Hasan Bitar, Anna K Zachrisson, Martin Byström, Joakim Strömberg

**Affiliations:** 1 University of Gothenburg, Sahlgrenska Academy, Institute of Clinical Sciences, Department of Hand Surgery, Gothenburg, Sweden; 2 Department of Surgery and Orthopaedics, Alingsås lasarett, Alingsås, Sweden; 3 Department of Surgery and Orthopaedics, Kungälvs sjukhus, Kungälv, Sweden; 4 Department of Hand Surgery, Sahlgrenska University Hospital, Mölndal, Sweden

**Keywords:** Trigger finger, trigger digit, trigger thumb, tendovaginitis stenosans, triggering, trigger phenomenon

## Abstract

This prospective randomized controlled study compared two injection techniques for trigger digit: either dorsal to the tendons in the proximal phalanx (PP group) or anterior to the tendons at the A1 pulley level (A1 group) in 106 patients. The primary outcome was the number of days to total relief of pain, stiffness and triggering, as recorded by the patients on visual analogue scales day-by-day for 6 weeks. The median number of days to complete symptom relief was 9 days in the PP group and 11 days in the A1 group for pain, 11 days and 15 days for stiffness and 21 and 20 days for triggering, respectively. Ninety-one per cent of all patients did not require any additional treatment, but 11 patients in both groups reported some remaining symptoms at 6 weeks. This study did not detect any significant difference between the two injection techniques, but provides detailed data of the rate and order of symptomatic relief after corticosteroid injection for this common condition.

**Level of evidence:** I

## Introduction

Corticosteroid injection is a well-established first-line treatment for trigger digit (Anderson and Kaye, 1991) and various techniques have been described. Taras et al. (1998) showed that injections in the subcutaneous tissue at the level of the A1 pulley were as efficient as intra-sheath deposition, and a parallel group study comparing three different techniques (Rosenbaum et al., 2018) did not detect any differences in regard to pain at the injection sites. There are however no randomized studies that compare different injection techniques. Furthermore, there are few studies that focus on the time aspect of symptom relief after this treatment. One study showed that triggering was resolved in 79% of the patients within a month and that the mean number of days to resolution of trigger phenomenon was 8.8 (Yak et al., 2020); another found that most patients experience relief of pain and triggering by 3 weeks after injection, with triggering lagging behind pain relief (Seigerman et al., 2021).

The rationale for this study was to compare two different techniques for corticosteroid injections in the tendon sheath: one at the level of the proximal phalanx (PP group) after bone contact and one at the A1 level (A1 group), with deposition of the corticosteroid either dorsal to (in the PP group) or anterior to (in the A1 group) the flexor tendons. Our hypothesis was that the former injection technique would be more precise and hence produce a more rapid alleviation of symptoms than the latter, and that it would be less painful since it did not affect the A1 region of the affected finger, which is usually tender. The study also investigated the time frame of resolution of the three most common symptoms of trigger digit, namely pain, stiffness and triggering, by daily recording on a visual analogue scale (VAS) by each patient.

## Methods

This was a prospective, multicentre, randomized parallel-group study conducted at three different hospitals between September 2020 and January 2022 on patients receiving their first ever corticosteroid injection for trigger digit. The patients were assessed and treated by two senior hand surgeons (JS and MB), one specialist in orthopaedic surgery (AZ) and one resident in orthopaedic surgery (HB). The levels of expertise of the participating surgeons according to Tang and Giddins (2016) were: JS 4, MB 3, AZ 2 and HB 1. The study was conducted according to the CONSORT (Consolidated Standards of Reporting Trials) guidelines (Altman et al., 2012) and registered at Clinical.Trials.gov (NCT04568993). The inclusion criteria in patients seeking treatment were trigger phenomenon in a single digit with stiffness or pain, or both, on movement, and tenderness over the metacarpophalangeal (MCP) joint of the affected digit. Only adult patients (>18 years) were included. Exclusion criteria were any other previous treatment or operation on the affected digit or any other pathological condition or limited range of motion the affected digit. Written informed consent was obtained from all patients.

The primary outcome measure was number of days to complete relief of pain, stiffness and triggering defined as ‘0’ on the VAS for each symptom. Secondary outcome measures were pain during injection, duration of local anaesthesia, pain after injection and two hand-specific questionnaires: the QuickDASH and the HQ-8 used in the Swedish Hand surgery register (Carlsson et al., 2021). Any additional procedures for trigger digit in the same digit during a 6-month period after the injection were recorded.

### Power analysis

A power analysis was done for the number of days to complete relief of symptom: an a priori sample-size estimate indicated that 43 patients were required in each group given a significance level (α) of 0.05 and a power (ß) of 0.85 for a minimum clinically important difference of 2 days to complete relief of symptoms between the two groups. Anticipating a loss to follow-up, we added ten patients to each group to give a total of 106 patients.

### Randomization procedures

Randomization was by sealed envelopes prepared by a secretary according to a randomization sequence generated by a computer (https://www.randomzier.org). The 106 envelopes were numbered and contained 53 notes that read ‘PP’ and 53 ‘A1’ according to the randomization sequence. The envelopes were divided between the participating clinics and opened after the patients had filled in the baseline questionnaires and the written consent.

### Baseline registration

Before treatment, the patients completed the QuickDASH and HQ-8 questionnaires along with self-assessment of pain, stiffness and frequency of triggering using VASs (range 0–10). The VAS endpoints were defined with the following anchor statements for each symptom: pain (0, ‘no pain’; 10, ‘worst imaginable pain’); stiffness (0, ‘no stiffness’; 10, ‘maximum stiffness’); and frequency of triggering (0, ‘none’; 10, ‘constantly’).

For both injections, 1 ml Depo-Medrone® with lidocaine (Pfizer, NY, USA), which is a mixture of methylprednisolone (40 mg/ml) and lidocaine (10 mg/ml), was injected using a 25-gauge needle attached to a 2.5 ml syringe.

### Injection in the proximal phalanx (PP group)

The injection site was located in the centre of the palmar aspect of the proximal phalanx in the midline between the flexion creases. The needle was inserted through skin, subcutaneous tissue, tendon sheath and tendon until bone was reached. Gentle pressure was applied on the syringe as it was retracted slightly to enable free flow of the corticosteroid.

### Injection at the A1 level (A1 group)

The injection site was located palmar to the MCP joint in the midline and centred over the A1 pulley. The needle was inserted through skin and subcutaneous tissue into the tendon. The location was confirmed by asking the patient to flex the finger slightly, causing movement of the syringe. If this did not occur, the needle was inserted deeper until it did. At this point, gentle pressure was applied on the syringe as it was retracted to enable free flow of the corticosteroid.

### Follow-up forms and procedures

The patients were discharged with follow-up forms for daily self-assessment of symptoms for the initial 4 weeks and an envelope with a prepaid stamp to facilitate the return of the completed forms. On injection day they were asked to rate their pain during and after injection on a VAS and also the duration of the local anaesthesia. The patients were instructed to contact the investigators by phone or email if any complication should arise.

During the following 4 weeks the patient graded their symptom severity on the VASs for pain, stiffness and frequency of trigger phenomenon on a daily basis until complete recovery (represented by 0 on the VAS), using the same anchor statements described for the baseline registration. The patients were told to send in their completed forms when they were fully satisfied with the injection treatment and also to complete the included QuickDASH and HQ-8 forms at this time. Patients who had not returned their forms by 3.5 weeks were contacted by phone by the investigators and sent a new follow-up form for another 2 weeks, after which all patients were instructed to return their forms regardless of any residual symptoms. Patients who were not satisfied (defined as VAS > 0 for any symptom) by 6 weeks were contacted by phone for further counselling or repeated treatment or both. The details of any patients that contacted the investigators within 6 months after the injection were also recorded.

### Statistics

Since all datasets failed the Shapiro–Wilk test for normal distribution, values were reported mainly as median (range or IQR) and non-parametric statistics were used throughout the analyses. The Mann–Whitney *U*-test was used to compare the PP and A1 groups for the number of days to symptomatic relief, pain and duration of anaesthesia and also the HQ-8 and QuickDASH scores. Cross tabulations and Fisher’s exact test were used to compare the number of patients who had residual symptoms after 6 weeks. Wilcoxon’s signed rank test was used to test changes in VAS, HQ-8 and QuickDASH scores at the baseline and after 6 weeks in each patient. To avoid the problem of mass significance when comparing day-to-day VAS records between the two treatments, linear regression coefficients were calculated for each patient and for each variable (pain, stiffness and trigger phenomenon), thus describing the trend of either treatment over time from 0 to 42 days. The differences were analysed using Fisher’s exact test.

## Results

Between September 2020 and January 2022, 434 patients with a trigger digit were referred to the three hospitals and 106 were enrolled. Fifty-three patients were randomized to injection at the A1 pulley level and 53 to injection in the proximal phalanx. The baseline characteristics of the patients were similar in the two groups (Table 1) except for the affected digits, with twice the number of thumbs in the A1 group than in the PP group. There were no adverse events from the injections, and all patients received the follow-up form after treatment.

**Table 1.** Baseline patient characteristics.

Variable	Proximal phalanx(*n = *48)	A1-pulley level(*n = *1)
Age (years)		
Median (min.–max.)	64 (31–81)	62 (40–84)
Gender, *n* (%)		
Male	14 (29%)	20 (39%)
Female	34 (71%)	31 (61%)
Digit involved, *n*		
Thumb	6	12
Index	4	2
Middle	16	17
Ring	20	18
Little	2	2
Symptoms		
Triggering	29	34
Tenderness over pulley	45	50
Concomitant disease, *n*		
Diabetes	7	10
Rheumatic disease	0	3
Duration since first symptoms (months)		
Median	6	8
Range (min.–max.)	1–36	1–48
VAS-score, median (min.–max.)		
Pain	6.6 (0–10)	6.4 (0–10)
Stiffness	7.4 (1–10)	8.0 (0–10)
Trigger phenomenon	5.4 (0–10)	5.0 (0–10)
QuickDASH score, median (min.–max.)	34 (5–73)	39 (0–68)
HQ-8 scores, median (min.–max.)		
Pain on load	70 (0–100)	70 (0–100)
2. Pain on motion without load	50 (0–90)	40 (0–90)
3. Pain at rest	30 (0–80)	30 (0–90)
4. Stiffness	70 (0–100)	70 (0–100)
5. Weakness	60 (0–100)	50 (0–100)
6. Numbness/tingling in fingers	10 (0–90)	10 (0–80)
7. Cold sensitivity	20 (0–100)	10 (0–100)
8. Ability to perform daily activities	50 (0–100)	50 (0–100)

VAS: visual analogue scale; min.–max.: minimum to maximum.

**Figure 1.**
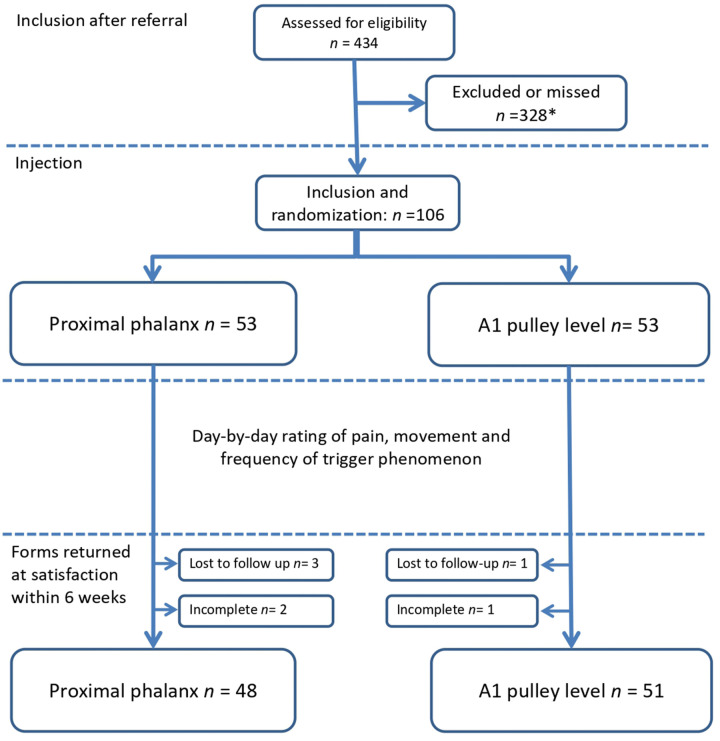
Flowchart of the study. * The number represents a retrospective analysis of all patients diagnosed with trigger digit in our three departments during the study period who were not enrolled in this study either because of exclusion criteria or were missed by the authors.

Three patients in the PP group and one in the A1 group were lost to follow-up; two patients in the PP group and one in the A1 group sent in incomplete forms, leaving 48 complete forms (91%) in the PP group and 51 complete forms (96%) in the A1 group for analysis (Figure 1). No complications after injection were reported and all patients with residual symptoms were contacted or assessed, or both, during a 6-month period after the injection.

The results are presented in Table 2. Most patients in both groups had complete relief of all symptoms within 6 weeks (77% in the PP group and 78% in the A1 group), while 11 patients in both groups reported VAS more than 0 for one or more symptom at 6 weeks. However, these patients had a reduction in symptom severity by at least 61% (range 61%–79%; Table 2). There were no significant differences between the two groups at any time regarding any outcome measure except for the duration of the local anaesthesia, but a tendency towards a more rapid relief of pain was noted in the PP group. Most patients in both groups were fully satisfied with the treatment and would choose to have another corticosteroid injection.

**Table 2.** Results at 6 weeks.

Variable	Proximal phalanx(*n = *48)	A1-pulley level(*n = *51)	*p-*value
Number of days to complete relief of symptoms*, median (range)			
Pain			
Median (min.–max.)	9 (1–29)	11 (1–37)	0.135
Stiffness			
Median (min.–max.)	11 (4–36)	15 (4–41)	0.135
Triggering of digit			
Median (min.–max.)	21 (4–35)	20 (1–41)	0.553
Patients with remaining symptoms at 6 weeks			
Pain			
Patients, *n* (%)	2 (4)	9 (16)	0.051
Relative VAS improvement from baseline, mean (SD)	77% (SD 9)	73% (SD 21)	
Stiffness			
Patients, *n* (%)	7 (15)	8 (16)	0.969
Relative VAS improvement from baseline, mean (SD)	66% (SD 28)	71% (SD 24)	
Triggering of digit			
Patients, *n* (%)	9 (19)	10 (20)	0.958
Relative VAS improvement from baseline, mean (SD)	61% (SD 35)	69% (SD 33)	
Pain at injection and duration of anaesthesia			
Patient rated pain (VAS)			
Median (min.–max.)	5.6 (0–9.8)	5.5 (0–9.9)	0.945
Patient rated duration anaesthesia (min)			
Median (min.–max.)	70 (20–360)	60 (20–270)	0.041
QuickDASH, median (min.–max.)			
Baseline	34 (5–73)	39 (0–68)	0.988
At 6 weeks or at satisfaction	4.5 (0–41)	4.5 (0–53)	
Improvement from baseline	–27 (7–68)	–26 (0–64)	
HQ-questionnaire: improvement from baseline, median (min.–max.)			
Pain on load	58 (0–100)	50 (–40–100)	0.157
2. Pain on motion without load	40 (–10 to 90)	40 (0–90)	0.750
3. Pain at rest	20 (–10 to 80)	30 (0–90)	0.402
4. Stiffness	55 (–20 to100)	50 (0–90)	0.278
5. Weakness	50 (–30 to 90)	40 (–10 to 100)	0.599
6. Numbness/tingling in fingers	0 (0-80)	5 (–10 to 60)	0.919
7. Cold sensitivity	10 (–10 to 100)	0 (–20 to 100)	0.376
8. Ability to perform daily activities	45 (–40 to100)	30 (–20 to 100)	0.838
Patient satisfaction and attitude towards repeated corticosteroid injection			
Patient-rated result of treatment^§^ (VAS)			
Median (min.–max.)	10 (1–10)	10 (4–10)	0.568
Patient’s attitude towards repeated corticosteroid injection^#^ (VAS)			
Median (min.–max.)	10 (5–10)	10 (5–10)	0.269

*In patients who reported symptom at baseline and complete relief within 6 weeks. Complete relief was defined as VAS score = 0.

§ The patient’s response to the question ‘How do you rate the result of the treatment?’ on a scale from 0 to 10, where was 0 defined as ‘totally dissatisfied’ and 10 ‘completely satisfied’.

# The patient’s response to the question ‘If you had the same symptoms again, how likely is it that you would choose the same treatment?’ on a scale from 0 to 10, where 0 was defined as ‘not likely at all’ and 10 ‘very likely’.

VAS: visual analogue scale; min.-max.: minimum to maximum.

Mean (SD) values are given when the number of patients in a group is small.

**Figure 2.**
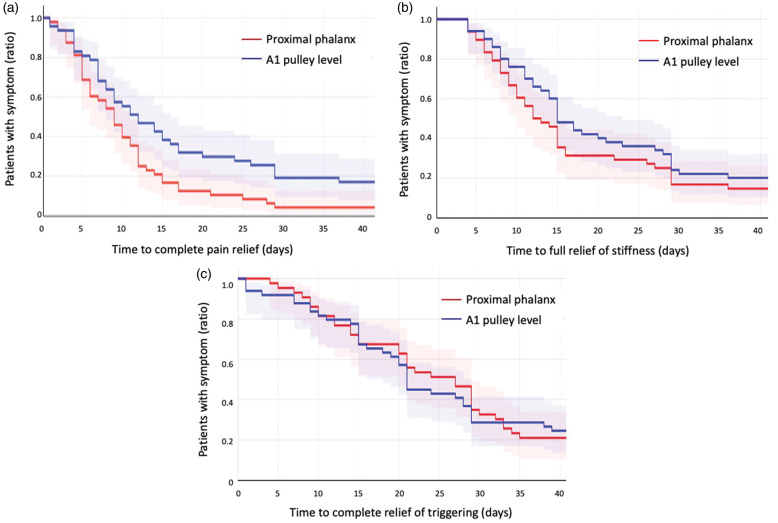
Kaplan–Meier survival plots with confidence intervals for (a) pain, (b) stiffness and (c) triggering. The terminal event was defined as complete relief of each symptom. The *y*-axis denotes the ratio of patients with remaining symptom and the *x*-axis the number of days after injection. For example, in 2(a), approximately 80% in the PP group and 60% in the A1 group were pain-free after 14 days.

**Figure 3.**
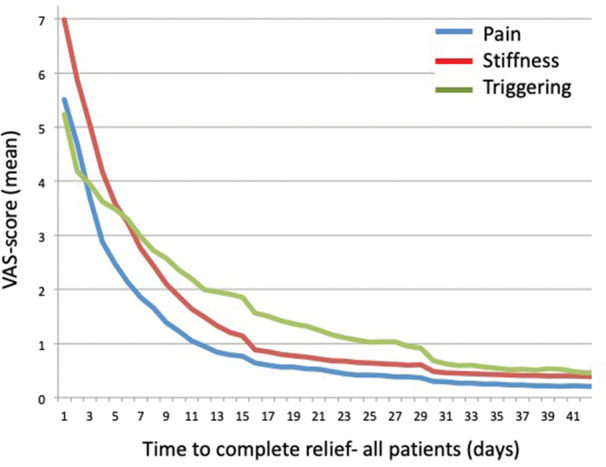
Mean visual analogue scale scores for all patients per day after injection.

The number of days to complete relief of each symptom are described by Kaplan–Meier plots for the relative number of patients in each group with remaining symptoms (Figure 2) per day. Patients in both groups recorded the same sequence of symptom relief: pain, stiffness and triggering (Figure 3). There were no significant differences between individual day-by-day VAS scores between the groups regarding pain (*p = *0.21), stiffness (*p > *0.30) and triggering (*p > *0.30) as analysed by linear regression coefficients.

Out of the 11 patients in each group who had remaining symptoms at 6 weeks, seven in the PP group and six in the A1 group, became satisfied without any further treatment; four in the PP group and two in the A1 group had a repeated injection; none of those in the PP group and three in the A1 group were scheduled for open release within 6 months.

## Discussion

This study did not detect any significant difference between the results after corticosteroid injection for trigger digit in the proximal phalanx and at the A1 pulley level, except for a longer duration of anaesthesia after injection in the proximal phalanx, which could be considered clinically irrelevant. However, the results indicate a tendency for injection in the proximal phalanx to provide a more rapid relief of the pain associated with trigger digit disease, both in regard to number of days to pain relief (Table 2), but also the rate at which this is accomplished (Figure 2(a)). This study confirmed that corticosteroid is an excellent first treatment option for this common condition: 91% of all patients did not require any further treatment within 6 months, there were no post-injection complications and most patients were fully satisfied and would choose a repeated injection if the symptoms recurred. Three patients in the A1 group but none in the PP group required open release within 6 months of injection. This might be a coincidence, and a long-term follow-up of the participants might show if this is a true difference in recurrence rates between the two injection techniques.

The sequence of relief of symptoms was similar after both injection techniques: pain seems to subside first, followed by improvement of stiffness while triggering seems to linger (Figure 3). These results are consistent with the findings by Seigerman et al. (2021), but the timing of symptom relief differed. While they found that 82% had complete improvement in pain and 65% triggering at 3 weeks, our corresponding results were 78% and 45%, respectively. Furthermore, Yak et al. (2020) reported resolution of triggering in 79% of the patients within a month, and our results are consistent with these findings (81% in the PP group and 81% in the A1 group). However, while they reported that the mean number of days to resolution was 8.8, the patients in our study reported a significantly longer duration (21 days in the PP group and 20 days in the A1 group). The reason for this difference could be that our study included more severe cases (Grade IV), but it could also be attributed to different methodologies, since they used a postcard with a prepaid stamp that the patients were instructed to mail when the triggering had resolved, whereas the patients in our study recorded day-by-day.

There are some limitations to this study. First, it was entirely based on the patients’ experiences since no objective follow-up measurements were done. The accuracy of the recordings by the patients cannot be verified, nor the timing of their completion of the forms. We simply had to assume that the patients who completed the forms did this according to instructions, namely day-by-day. As discussed by Graham et al. (2015), satisfaction measures in musculoskeletal diseases are complex and even though the VAS is widely used beyond its original use as a pain measure, its merits have been questioned. Badalamente et al. (2013) highlight two possible deficiencies of the VAS: the assumption that pain is linear and the notion that patients use essentially the same scaling for their symptoms. They conclude, however, that the VAS for pain has been shown to have good intrarater reliability and another study by Clark et al. (2003) showed that it is useful in reflecting change in the symptom over time in an individual. In this study, we believe that the change in VAS in the individual patient for either pain, stiffness and trigger phenomenon mirrors the alleviation of that specific symptom from baseline to complete relief, and that the rate in each patient can be described by the day-by-day responses. However, since this methodology has not been previously used to our knowledge, we chose the primary outcome accordingly, assuming that a patient would know with certainty when a symptom that was the reason for the injection had disappeared. Another limitation is that the restriction of the form to 6 weeks led to incomplete records of the exact details of the 22 patients who still had symptoms at this time.

The tendency for PP injections to lead to faster recovery than injections in the A1 region might be attributed to whether or not the corticosteroid was deposited in the tendon sheath. It could be hypothesized that the A1 technique is more dependent on the position of the needle, and that some of the patients in this group inadvertently received a subcutaneous injection. Regardless of the injection method, proximity to the tendon sheath or the A1 pulley during injection for trigger digit might be adequate: Jimenez et al. (2020) showed in a cadaveric study that even dorsal injections in the webspace will reach these structures. Roberts et al. (2021) retrospectively analysed 210 patient charts and found no significant differences between injection methods, but only between different corticosteroids. However, patients injected with methylprednisolone had subsequent open releases earlier than those injected with dexamethasone, indicating that the choice of corticosteroid should also be considered.

The question whether there is an optimal technique to inject corticosteroid is yet to be resolved. This study, however, provided new detailed knowledge about how patients rate their improvement day-by-day to complete symptom relief after injection by two of these techniques.

## Trial registration

Clinical.Trials.gov: NCT04568993.
